# Moisturizing Effects of Alcalase Hydrolysate Fractions from *Haliotis discus* Viscera, a Marine Organism, on Human Dermal Fibroblasts, HaCaT Keratinocytes, and Reconstructed Human Skin Tissues

**DOI:** 10.3390/md22110503

**Published:** 2024-11-06

**Authors:** Nalae Kang, Eun-A Kim, Seong-Yeong Heo, Jun-Ho Heo, Ginnae Ahn, Soo-Jin Heo

**Affiliations:** 1 Jeju Bio Research Center, Korea Institute of Ocean Science and Technology (KIOST), Jeju 63349, Republic of Korea; nalae1207@kiost.ac.kr (N.K.); euna0718@kiost.ac.kr (E.-A.K.); syheo@kiost.ac.kr (S.-Y.H.); unknown0713@kiost.ac.kr (J.-H.H.); 2 Department of Food Technology and Nutrition, Chonnam National University, Yeosu 59626, Republic of Korea; gnahn@jnu.ac.kr; 3 Department of Biology, University of Science and Technology (UST), Daejeon 34113, Republic of Korea

**Keywords:** *Haliotis discus*, viscera, hydrolysate, moisturizing effect, cosmeceuticals, HDF, HaCaT, reconstructed human skin tissue

## Abstract

*Haliotis discus*, an abalone, is a marine gastropod mollusk that has been cultivated globally owing to its nutritional value and high market demand. However, the visceral parts of *H. discus* are typically discarded as by-products, highlighting the need to explore their potential value in developing cosmeceuticals and pharmaceuticals. This study investigated the potential moisturizing effects of *H. discus* visceral tissues. Various hydrolysates from *H. discus* viscera tissue were evaluated for proximate composition, radical scavenging, and hyaluronidase inhibition activities. Alcalase hydrolysate was isolated using gel filtration chromatography (GFC), and its moisturizing effects were tested on human dermal fibroblasts (HDF), HaCaT keratinocytes, and reconstructed human skin tissue. The Alcalase hydrolysate showed the highest extraction yield, radical scavenging, and hyaluronidase inhibition activities. The Alcalase hydrolysate GFC fraction 1 increased collagen synthesis-related molecules, including procollagen type 1 in HDF and hyaluronic acid-related molecules in HaCaT cells. These moisturizing effects were confirmed in reconstructed human skin tissues by increased levels of aquaporin 3 and filaggrin. Fraction 1 consisted of two main peptides: DNPLLPGPPF and SADNPLLPGPPF. In conclusion, *H. discus* Alcalase hydrolysate and its fractions have potential moisturizing properties and can be used as cosmeceuticals.

## 1. Introduction

Human skin, the body’s largest organ, performs multiple functions, including sensation, heat regulation, and water conservation. It also influences individual appearance and identity [1,2]. Constant exposure to environmental factors such as solar ultraviolet radiation, visible light, and pollutants causes the skin more stress than most organs, contributing to early signs of aging, such as skin dehydration [2]. Skin hydration is critical for maintaining healthy skin, and moisturizers are essential components of basic skin care [3].

The skin is composed of two main layers, the epidermis and dermis, each exhibiting unique structural and physiological functions. As the epidermis is directly exposed to the external environment, its primary function is to serve as a barrier and is mainly composed of keratinocytes [4]. Keratinocytes are arranged in layers throughout the epidermis; as these cells divide and proliferate away from the basal layer, which is closest to the dermis, they begin to differentiate [4,5]. The dermis, located beneath the epidermis, is primarily composed of complex extracellular matrix (ECM) proteins, including collagen, which provide structural support. Fibroblasts in the dermis synthesize extracellular matrix components, including collagen fibers [5]. 

The term “cosmeceutical” refers to a cosmetic product exhibiting drug-like properties. This blending of cosmetics and pharmaceuticals indicates the growing convergence of these two fields [6]. Increasing interest in skin health and advances in understanding skin structure, physiology, and aging have led to the identification of novel biomarkers of skin health, including ECM, hyaluronic acid, aquaporin, and filaggrin. Chemical manipulation of these targets has the potential to facilitate the restoration and maintenance of healthy skin [7]. In recent decades, demand for cosmeceutical ingredients derived from natural products has considerably increased. The search for novel natural ingredients in the cosmetics industry has led to the accumulation of diverse plant materials from various geographical regions. These include flowers, seeds, roots, leaves, twigs, and berries sourced from various plant species [7]. The marine environment, rich in macroorganisms and microorganisms, has evolved distinctive metabolic adaptations for survival in diverse, challenging habitats. This has led to the synthesis of numerous secondary metabolites with distinct functionalities, many of which are commercially valuable in the pharmaceutical and cosmeceutical industries [8].

*Haliotis discus*, a species of abalone, is a marine gastropod mollusk that inhabits the intertidal and subtidal zones of tropical and temperate coasts [9]. *H. discus* has been the most harvested and studied species globally owing to its nutritional value and high market demand [10]. Abalone has numerous biological properties, including antioxidant [11], anti-inflammatory [12], osteogenic [13], and immunostimulatory [14] activities. In particular, the visceral parts of abalone exhibit antioxidant [15], anti-obesity [16], and angiotensin-converting enzyme inhibitory activities [17]. However, viscera accounting for 15–25% of the total weight of the abalone are typically discarded as by-products, constituting a substantial economic burden for producers [18,19]. Therefore, it is imperative to identify the potential value of abalone viscera generated during fishing processing and investigate its use in developing cosmeceuticals and pharmaceuticals.

To explore the potential use of *H. discus*, a marine mollusk, for cosmeceutical, we investigated its moisturizing effects on human dermal fibroblasts (HDF) and HaCaT keratinocytes, two skin cell types. Finally, we confirmed these moisturizing effects on reconstructed human skin tissue, an advanced in vitro model. 

## 2. Results and Discussion

### 2.1. Extraction Yield and Proximate Composition of H. discus Viscera Hydrolysates

Enzymatic hydrolysis is a method used to produce bioactive peptides by cleaving peptide bonds in proteins [20]. Proteins are a major component of the viscera of marine mollusks [21]. Thus, *H. discus* viscera was hydrolyzed using four commercial food-grade proteases, including Alcalase, bromelain, Flavourzyme, and Neutrase, to extract a variety of potential bioactive properties from the proteins. These proteases have been applied to functional ingredients derived from marine animals [15,20,21], and each protease has different enzymatic characteristics: Alcalase, a serine endopeptidase; Bromelain, a mixture of different thiol endopeptidases and other components; Flavourzyme, a mixture of exo- and endopeptidases; Neutrase, a zinc metalloendo-protease [22,23,24,25]. The extraction yields and proximate compositions of the four *H. discus* hydrolysates are summarized in Table 1. All hydrolysates had >70% extraction yield and >30% protein content, whereas polysaccharide and total phenolic contents were relatively low. Notably, the Alcalase hydrolysate showed the highest extraction yield of 88.01 ± 0.53% and a protein content of 38.77 ± 0.45%.

### 2.2. Antioxidant and Hyaluronidase Inhibition Activities of H. discus Viscera Hydrolysates

Reactive oxygen species and oxidative stress in the skin can be induced by factors such as solar ultraviolet radiation, visible light, pollutants, and psychological stress [26,27]. Oxidative stress also contributes to skin aging, including dermal–epidermal junction flattening, reduced skin barrier function, and increased transepidermal water loss [28]. Thus, several studies have evaluated radical scavenging and hyaluronidase inhibitory activities to confirm the skin health effects of the candidates [29,30,31].

**Figure 1:**
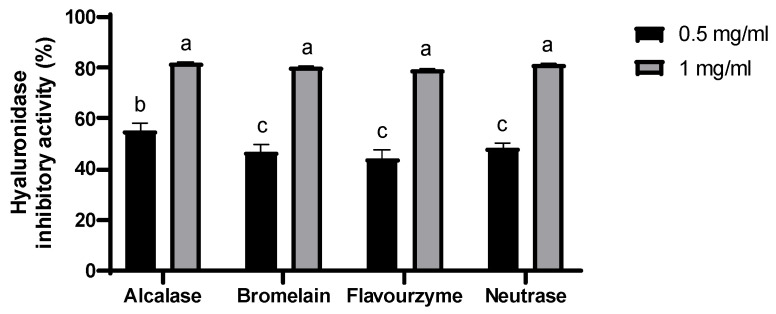
Hyaluronidase inhibition activity of four hydrolysates from *H. discus* viscera. Values are expressed as the mean ± SD of triplicate experiments. Different lowercase letters indicate significant differences for each experiment.

The antioxidant and hyaluronidase inhibitory activities of *H. discus* viscera hydrolysates are shown in Table 2 and Figure 1. Alcalase and Flavourzyme hydrolysates showed higher 2,2-diphenyl-1-picrylhydrazyl (DPPH) radical scavenging activity than bromelain and Neutrase hydrolysates. Alcalase hydrolysate showed the highest hydrogen peroxide scavenging activity, with a half-maximal inhibitory concentration (IC_50_) of 0.36 ± 0.07 mg/mL. Additionally, Alcalase and Neutrase hydrolysates exhibited the highest 2,2′-azino-bis(3-ethylbenzothiazoline-6-sulfonic acid) (ABTS) radical scavenging activity with IC_50_ values of 0.31 ± 0.00 and 0.31 ± 0.01 mg/mL, respectively. In particular, Alcalase hydrolysate exhibited the highest hyaluronidase inhibition activity, with approximately 55% inhibition at 0.5 mg/mL. Based on activity, extraction yield, and protein content, the viscera Alcalase hydrolysate of *H. discus* was selected for further isolation experiments.

### 2.3. Isolation of Bioactive Properties from H. discus Viscera Alcalase Hydrolysates

**Figure 2:**
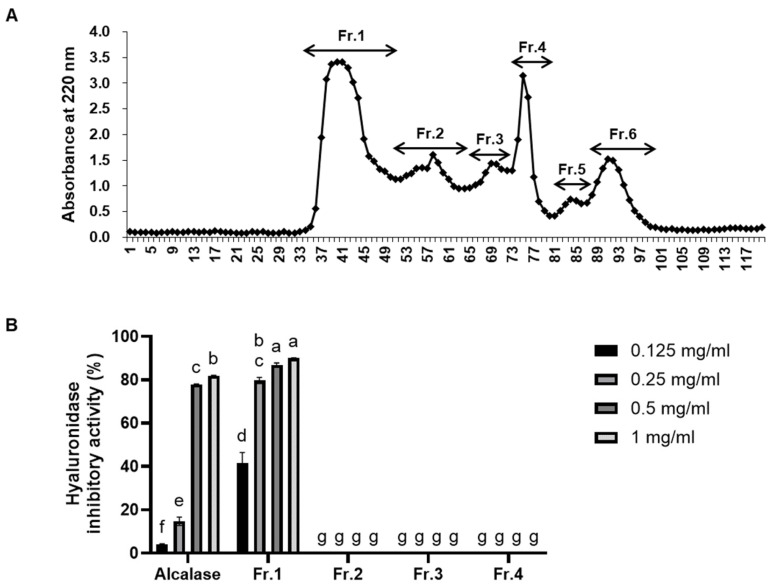
Hyaluronidase inhibition activities of Alcalase hydrolysates gel filtration chromatography fractions. (**A**) Gel filtration chromatogram of Alcalase hydrolysates using Sephadex G-25. (**B**) Hyaluronidase inhibition activities for each fraction. Values are expressed as the mean ± SD of triplicate experiments. Different lowercase letters indicate significant differences for each experiment.

Gel filtration chromatography is a technique used to separate protein enzymatic hydrolysates based on molecular size [32,33]. The Alcalase hydrolysate was fractionated into six fractions (Figure 2A). Among these, only fraction 1 (Al-Fr.1) exhibited higher hyaluronidase inhibition activity than the Alcalase hydrolysate (Figure 2B). 

### 2.4. Effect of Al-Fr.1 on Collagen Synthesis in Human Dermal Fibroblasts

**Figure 3:**
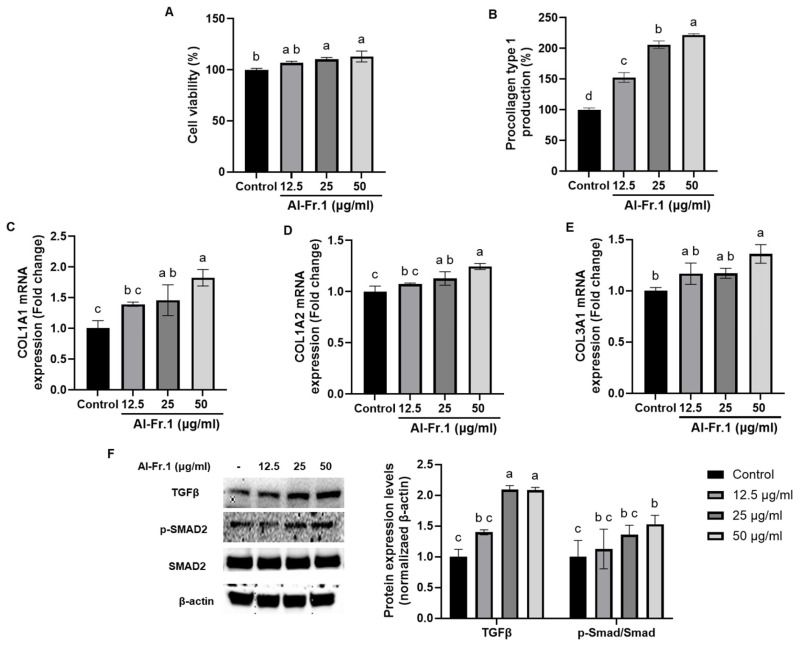
Collagen synthesis effects of Al-Fr.1 via the TGFβ/ SMAD signaling pathway in HDF. Cells were treated with different Al-Fr.1 concentrations (12.5, 25, and 50 µg/mL) for 24 h. (**A**) Cell viability was measured using an MTT assay. (**B**) Procollagen type 1 production levels were measured in supernatants using the Procollagen Type 1 C-peptide Kit. (**C**–**E**) mRNA levels of COL1A1, COL1A2, and COL3A1 were analyzed using qPCR. (**F**) Protein levels of TGFβ, p-SMAD2, SMAD2, and β-actin were analyzed using Western blot analysis. Quantitative analysis was performed using ImageJ 1.54k software. Values are expressed as the mean ± SD of triplicate experiments. Different lowercase letters indicate significant differences.

Dermal fibroblasts synthesize and organize the ECM, including collagens (type 1 and type 3 collagens, accounting for approximately 95%), which are essential components of dermal tissue [34,35]. Collagen provides tensile strength and maintains cellular structure; thus, a decrease in the number and length of collagen fibers in fibroblasts reduces dermal elasticity, causing deep wrinkles and dryness of the epidermal layer [36]. Thus, we evaluated the effect of Al-Fr.1 on collagen synthesis in HDF (Figure 3). No cytotoxicity was observed for Al-Fr.1 at concentrations of 12.5, 25, and 50 μg/mL in HDF, and cell proliferation was noted at 25 and 50 μg/mL (Figure 3A). Al-Fr.1 increased procollagen type 1 levels in a concentration-dependent manner, with a twofold increase at 50 μg/mL compared to the control (Figure 3B). Al-Fr.1 also significantly upregulated the transcription of Collagen Type I Alpha 1 chain (COL1A1), COL1A2, and COL3A1, the primary collagen isotypes produced by fibroblasts, in a concentration-dependent manner (Figure 3C–E). Transforming growth factor (TGF)-β, a major pro-fibrogenic growth factor, induces SMAD2 to bind to the cytoplasmic receptor domain, leading to serine residue phosphorylation. The phosphorylated SMAD2 subsequently translocates to the nucleus and binds to its promoter sequence, activating procollagen synthesis [37]. Al-Fr.1 increased TGF-β expression and SMAD2 phosphorylation levels compared to the control. Therefore, our findings indicated that Al-Fr.1 upregulates collagen synthesis in HDF cells via the TGF-β/SMAD pathway.

### 2.5. Moisturizing Effect of Al-Fr.1 on HaCaT Keratinocytes

**Figure 4:**
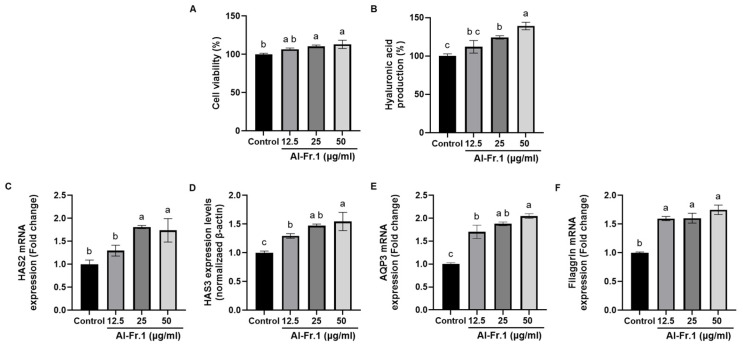
Moisturizing effect of Al-Fr.1 in HaCaT cells. Cells were treated with different Al-Fr.1 concentrations (12.5, 25, and 50 µg/mL) for 24 h. (**A**) Cell viability was measured using the MTT assay. (**B**) Hyaluronic acid production levels were measured in supernatants using the hyaluronan Kit. (**C**–**F**) mRNA levels of HAS2, HAS3, AQP3, and filaggrin were analyzed using qPCR. Values are expressed as the mean ± SD of triplicate experiments. Different lowercase letters indicate significant differences.

Hyaluronic acid (HA), an anionic glycosaminoglycan, is a key component of the ECM in basal keratinocytes, synthesized by HA synthase (HAS), and plays a role in skin moisturizing [38,39,40]. Therefore, the effect of Al-Fr.1 on HA synthesis was evaluated in HaCaT keratinocytes. Al-Fr.1 showed no cytotoxicity at 12.5, 25, and 50 μg/mL and induced cell proliferation at 25 and 50 μg/mL (Figure 4A), similar to fibroblasts. Al-Fr.1 significantly increased HA levels at all tested concentrations (12.5, 25, and 50 μg/mL) (Figure 4B). Al-Fr.1 also upregulated the transcription of HAS2 and HAS3, membrane-associated enzymes responsible for HA synthesis (Figure 4C,D). As HAS2 and HAS3 are the most abundant isotypes in keratinocytes [40,41], these results suggested that Al-Fr.1 induces HA synthesis.

Aquaporins 3 (AQP3) and filaggrin are key moisturizing factors involved in epidermal homeostasis. Aquaporins are a ubiquitous family of membrane proteins that maintain water homeostasis in all living cells by facilitating rapid water transport across cell membranes [42]. Filaggrin, found in the stratum corneum (the outer layer of the epidermis), contributes to water retention by incorporation into the lipid envelope or releasing free amino acids [43,44]. As shown in Figure 4E,F, Al-Fr.1 increased the transcription levels of AQP3 and filaggrin in HaCaT keratinocytes. Thus, these results indicated that Al-Fr.1 possesses a moisturizing effect by synthesizing HA and upregulating AQP3 and filaggrin in keratinocytes. 

### 2.6. Moisturizing Effect of Al-Fr.1 via the Mitogen-Activated Protein Kinase Signaling Pathway in HaCaT Keratinocytes

**Figure 5:**
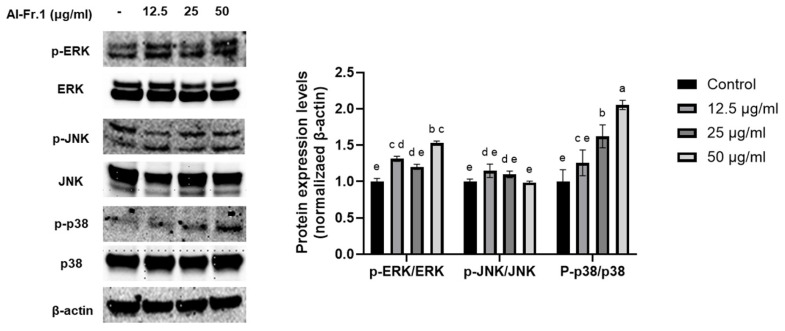
Effect of Al-Fr.1 via the MAPK signaling pathway in HaCaT cells. Cells were treated with Al-Fr.1 at concentrations of 12.5, 25, and 50 µg/mL for 24 h. Protein levels of p-ERK, ERK, p-JNK, JNK, p-p38, p38, and β-actin were analyzed using Western blot analysis. Quantitative analysis was performed using ImageJ software. Values are expressed as the mean ± SD of triplicate experiments. Different lowercase letters indicate significant differences.

The mitogen-activated protein kinase (MAPK) signaling pathway is involved in several cellular functions, including cell growth, antioxidant activity, and inflammation [45,46]. To analyze the relationship between the moisturizing effect of Al-Fr.1 and MAPK signaling, the expression and phosphorylation levels of proteins, including extracellular signal-regulated kinases (ERK), c-Jun NH2-terminal protein kinases (JNK), and p38, were measured in HaCaT keratinocytes (Figure 5). Al-Fr.1 induced phosphorylation of both ERK and p38 compared with the control group, with a notable increase in p38 phosphorylation. In contrast, Al-Fr.1 did not influence JNK phosphorylation levels. A relationship between skin hydration and the MAPK signaling pathway has been reported. Activation of the ERK signaling pathway is important for increasing HAS expression [40]. Protopanaxatriol, a secondary metabolite of ginsenosides, upregulates filaggrin and HAS expression, which is blocked by MAPK inhibitors [47]. Therefore, our findings indicated that Al-Fr.1 possesses a moisturizing effect via the ERK and p38 pathways in HaCaT keratinocytes.

### 2.7. Moisturizing Effect of Al-Fr.1 in Reconstructed Human Skin Tissues

The Three Rules (3Rs: Replacement, Reduction, and Refinement), established in 1959, guide the ethical use of animals in product testing and scientific research [48]. Animal testing is controversial owing to the discomfort and pain experienced by animals, whether mild or severe. Furthermore, interspecies differences raise doubts about the reliability of animal testing for predicting human outcomes [49]. The European Commission and other regulatory bodies worldwide have banned animal testing for cosmetics. Research is actively developing alternative methods to replace animal testing in line with the ‘3R’s Principle’ [50].

**Figure 6:**
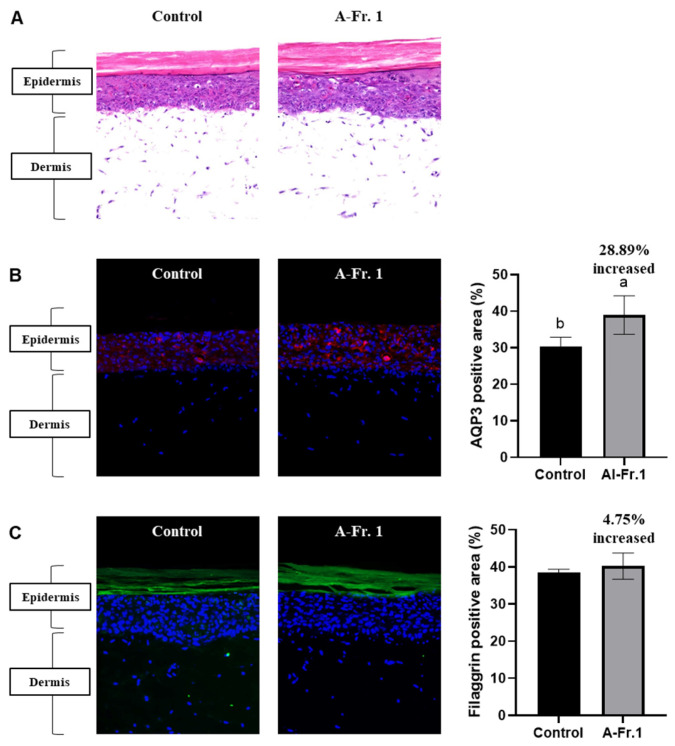
Moisturizing effect of Al-Fr.1 in human artificial skin tissues. Skin tissues were treated with Al-Fr.1 for 5 d, and tissue sections underwent H&E and immunofluorescence staining. (**A**) Morphology of the human artificial skin tissues. Comparison of AQP3 (**B**) and filaggrin (**C**) expression in human artificial skin tissues. Quantitative fluorescence analysis was performed using ImageJ software. Values are expressed as the mean ± SD of triplicate experiments. Different lowercase letters indicate significant differences.

The application of a reconstructed human skin model, comprising a well-formed epidermis and dermis, offers a promising alternative approach for evaluating cosmeceutical candidates [51]. The moisturizing effect of Al-Fr.1 was confirmed by measuring AQP3 and filaggrin expression in reconstructed human skin tissues using hematoxylin and eosin (H&E) and immunofluorescence staining techniques (Figure 6). The reconstructed human skin tissues used demonstrated general morphology, with a well-formed epidermis and dermis. The epidermis formed a hard stratum corneum and several other layers, and the dermis formed collagen fibrils around the fibroblasts (Figure 6A). Al-Fr.1 increased protein expression levels of AQP3 by 28.89% compared with the control in both the epidermis and dermis, excluding the stratum corneum (Figure 6B). Al-Fr.1 also increased the protein expression levels of filaggrin from the stratum granulosum to the stratum corneum of the epidermis by 4.75% compared to the control; however, this difference was not significant (Figure 6C). These results indicated that Al-Fr.1 enhances skin moisturization by regulating AQP3 and filaggrin expression levels, suggesting that reconstructed skin tissues are advanced in vitro models that complement cell-based experimental techniques.

### 2.8. Peptide Profiling of Al-Fr.1

**Figure 7:**
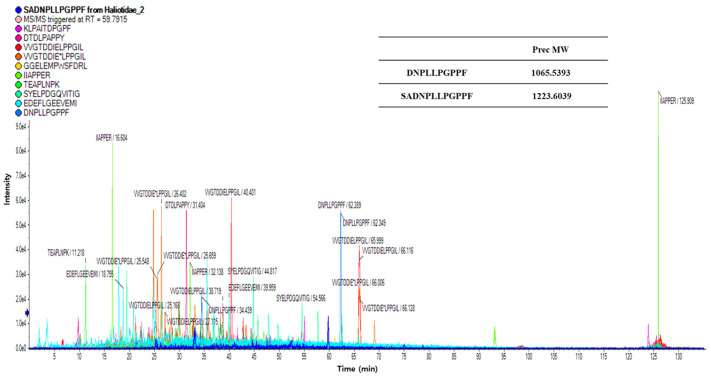
Two main peptides from Al-Fr.1.

The peptide profiling of Al-Fr.1 is shown in Figure 7. Seven peptides > 1000 Da were detected (KLPAITDPGPF, VVGTDDIELPPGIL, VVGTDDIE*LPPGIL, GGELEMPWSFDRL, SYELPDGQVITIG, EDEFLGEEVEMI, DNPLLPGPPF, and SADNPLLPGPPF) in Al-Fr.1. Notably, DNPLLPGPPF and SADNPLLPGPPF were repeatedly detected and thus identified as indicator peptides for Alcalase-Fr.1.

Marine organisms synthesize a diverse range of secondary metabolites to adapt to various ocean environments, including high salinity, high pressure, low hypoxia, and low light, making them attractive sources of novel functional ingredients [52]. However, sustainability is a critical consideration when developing such ingredients from marine resources. Marine by-products offer an environmentally friendly and sustainable source of materials [53,54]. 

In 2019, the production of *H. discus* reached 190,000 tons in China and South Korea, and the viscera, which accounts for approximately 15–25% of total weight, is often discarded as a by-product, contributing to environmental pollution and wasting economically valuable resources [34]. Using abalone viscera is an effective eco-friendly strategy for expanding the cosmeceutical industry. Hydrolysate fractions and peptides derived from *H. discus* viscera have the potential to be sustainable materials for cosmeceutical product development. 

To enhance the technology utilizing marine by-products, including abalone, comprehensive research should be conducted in relation to both food nutrition and medicinal applications, extending beyond cosmeceuticals. A substantial body of prior research has been conducted on functional ingredients derived from molluscan viscera [15,16,17]. *H. discus* feeds on seaweeds containing various antioxidants, which are used in cosmeceuticals. However, there are currently no direct studies on the relationship between diet and the peptide profiling and/or bioactivity of the viscera. Moreover, further investigation is necessary to ascertain whether the moisturizing effects are also exhibited with dietary intake of abalone. This should include additional research on hydrolysates using digestive enzymes such as pepsin and trypsin, as well as an analysis of the potential interaction with the body (Absorption, Distribution, Metabolism, Excretion, Toxicity).

## 3. Materials and Methods

### 3.1. Materials

Abalone (*H. discus*) was cultivated on a commercial scale at a fish farm in Wando, South Korea, and subsequently purchased from a fishing village market. After separating the shell, muscle, and viscera, visceral tissue was carefully washed three times with tap water and stored at −20 °C. Visceral tissues were freeze-dried and finely ground before hydrolysis. Commercial proteases, including Alcalase 2.4 L FG, Flavourzyme 500 MG, and Neutrase 0.8 L, were purchased from Novozyme Nordisk (Bagasvaerd, Denmark). Bromelain was purchased from Sigma-Aldrich (St. Louis, MO, USA). All other chemicals and reagents used were of analytical grade.

### 3.2. Preparation of H. discus Viscera Hydrolysates

Hydrolysates of *H. discus* viscera were prepared according to the method described by Kang et al. [21]. Two grams of *H. discus* viscera powder was hydrolyzed in 100 mL of buffer with hydrolytic protease in a substrate/enzyme ratio of 100:1 (*w*/*w*). Enzymatic hydrolysis was performed for 24 h under optimal temperature and pH conditions: Alcalase (50 °C, pH 8), bromelain (50 °C, pH 7), Flavourzyme (50 °C, pH 7), and Neutrase (50 °C, pH 6). Each hydrolysate was boiled for 10 min at 95 °C to inactivate the enzyme and subsequently centrifugated at 3200 rpm and 20 min at 4 °C to separate the residue. All hydrolysates were freeze-dried and stored at −20 °C. The yields of each *H. discus* viscera hydrolysate were calculated as the percentage of dry weight relative to the initial sample weight. 

### 3.3. Chemical Composition of H. discus Viscera Hydrolysates

The protein content was analyzed using the bicinchoninic acid (BCA) protein assay Kit (Thermo Scientific, Waltham, MA, USA). Total polysaccharide content was analyzed using the phenol-sulfuric acid method [55], and total polyphenolic content was measured using the Folin–Ciocalteu method [56]. Each assay used bovine serum albumin, glucose, and gallic acid as reference standards.

### 3.4. Radical Scavenging Activities of H. discus Viscera Hydrolysates

Each hydrolysate of *H. discus* viscera was dissolved in distilled water at various concentrations for radical scavenging assays. DPPH radical, hydrogen peroxide and ABTS scavenging assays were performed using modified methods from Blois [57], Kim et al. [58], and Muller [59], respectively. The IC_50_ of each hydrolysate was calculated for comparison.

### 3.5. Hyaluronidase Inhibition Activity of H. discus Viscera Hydrolysates and Its Fractions

Each hydrolysate of *H. discus* viscera and its fractions were dissolved in distilled water at various concentrations for the hyaluronidase inhibition activity assay. The hyaluronidase inhibitory effect was evaluated according to the method described by Jiratchayamaethasakul et al. (2020) [30]. Samples and hyaluronidase in 0.1 M of acetate buffer (pH 3.6) were combined in a test tube and incubated at 37 °C for 20 min. A 12.5 mM calcium chloride was added to the mixture, followed by another incubation at 37 °C for 20 min. The activated mixture was treated with 2.4 mg/mL HA in 0.1 M acetate buffer (pH 3.6) and incubated at 37 °C for 40 min. Thereafter, 0.4N sodium hydroxide and 0.4N potassium tetraborate tetra-hydrated were added and incubated in a water bath at 100 °C for 3 min. After cooling at 25 °C, a 10 mg/mL 4-(Dimethylamino)benzaldehyde solution (composed of 35 mL acetic acid and 5 mL of 10N hydrochloric acid) was added to the mixture and incubated at room temperature for 20 min. Absorbance was measured at 585 nm (Multiskan Go, Thermo Scientific).

### 3.6. Separation of Potential Bioactive Compounds via Gel Filtration Chromatography

Hydrolysates of *H. discus* viscera were separated by molecular size using gel filtration chromatography, as previously described by Kang et al. [21]. The hydrolysate was dissolved in distilled water, loaded onto a Sephadex G-25 gel filtration column (2.5 × 75 cm), and equilibrated with distilled water. The column was eluted with distilled water at a flow rate of 1.0 mL/min. The eluted samples were collected at 5 mL/tube, and elution peaks were detected at 220 nm.

### 3.7. Cells and Cell Culture

HDF (PCS-201-012) was purchased from the American Type Culture Collection (Manassas, VA, USA) and cultured for 5–9 passages. Dulbecco’s modified Eagle’s medium (DMEM)/Nutrient Mixture F-12 (DMEM/F-12) mixed at a ratio of 3:1, supplemented with 10% fetal bovine serum (FBS) and 1% penicillin/streptomycin, was used for HDF maintenance. HaCaT keratinocytes (CLS300493) were purchased from Cytion (Eppelheim, Germany) and cultured for 35–45 passages. DMEM supplemented with 10% FBS and 1% penicillin/streptomycin was used to maintain HaCaT keratinocytes. These cells were incubated at 37 °C in a 5% CO_2_ humidified atmosphere.

### 3.8. Cytotoxicity

HDF (2 × 10^4^ cells/well in a 96-well plate) and HaCaT keratinocytes (2 × 10^5^ cells/well in a 24-well plate) were seeded. After 16 h of incubation, cells were treated with various concentrations of test samples (12.5, 25, and 50 μg/mL) for 24 h. The cell viability was measured using the 3-(4,5-dimethylthiazol-2-yl)-2,5-diphenyltetrasolium bromide (MTT) (Invitrogen, Waltham, MA, USA) assay [60]. 

### 3.9. Sample Treatment

HDF (2 × 10^4^ cells/well in a 96-well plate) and HaCaT keratinocytes (2 × 10^5^ cells/well in a 24-well plate) were seeded and incubated for 16 h. After washing twice with Dulbecco’s Phosphate Buffered Saline (DPBS), new serum-free media (DMEM and DMEM/F-12 at a ratio of 3:1 with 1% penicillin/streptomycin) was added to the cells. Thereafter, cells were treated with various concentrations of test samples (12.5, 25, and 50 μg/mL) for 24 h, and each supernatant media and protein were collected for the next experiments.

### 3.10. Enzyme-Linked Immunosorbent Assay 

HDF and HaCaT keratinocytes treated with the test samples were prepared as described in Section 3.9. Procollagen type 1 and HA levels were measured using the enzyme-linked immunosorbent assay with the Procollagen Type I C-peptide Kit (Takara Bio Inc. Shiga, Japan) and hyaluronan assay Kit (R&D Systems, Minneapolis, MN, USA), respectively, according to the manufacturer’s instructions. Procollagen type 1 levels were measured in the supernatant media of HDF, and HA levels were measured in the supernatant media of HaCaT keratinocytes. 

### 3.11. Quantitative Polymerase Chain Reaction (qPCR) 

HDF and HaCaT keratinocytes treated with the test samples were prepared as described in Section 3.9. Thereafter, their mRNA levels were assessed. The qPCR was performed according to the method described by Kang et al. [60]. Total RNA was isolated using the TRIzol reagent (Invitrogen), and complementary DNA (cDNA) was synthesized from 2 μg of total RNA using a High-Capacity RNA-to-cDNA Kit (Applied Biosystems, Waltham, MT, USA). qPCR was performed using the Power SYBR Green PCR Master Mix (Applied Biosystems) on a QuantStudio 3 Real-Time PCR System (Thermo Fisher Scientific). Primers used in the present study are listed in Table 3.

### 3.12. Western Blotting

HDF and HaCaT keratinocytes treated with test samples were prepared as described in Section 3.9 and assessed for protein expression levels. Western blotting was performed following the method described by Kim et al. [34]. The cells were extracted using RIPA cell lysis buffer (1×) with EDTA (R4100-010, GenDEPOT, Katy, TX, USA), and the protein concentrations in the cell lysates were measured using the BCA Protein Assay Kit (Thermo Scientific). The same concentrated proteins were separated by Bolt^TM^ 12% Bis-Tris Plus Gels electrophoresis and transferred to nitrocellulose membranes (iBlot 2 NC regular Stacks) using an iBlot 2 gel transfer machine (Invitrogen). The membranes were then blocked with a mixture of 3% BSA (A7906, Sigma-Aldrich) and 2% skim milk (232100, BD, Franklin Lakes, NJ, USA) in tris-buffered saline (TBS) containing 0.1% tween 20 (TBST) for 1.5 h. The membranes were washed with TBST three times, incubated with primary antibodies in Pierce^TM^ Clear Milk Blocking Buffer (Thermo Scientific) overnight at 4 °C, washed with TBST three times, incubated with appropriate secondary antibodies, and washed again. The primary antibodies against TGFβ (3711), p-smad2 (8828), smad2 (8685), ERK (4695), p-JNK (9251), JNK (9252), p-p38 (4511), and p38 (9690) were obtained from Cell Signaling Technology (Beverly, MA, USA), and p-ERK (sc-7383) and β-actin (sc-47778) were purchased from Santacruz (Dallas, TX, USA), and were diluted at a 1:1000 ratio. The secondary antibodies anti-mouse IgG (G21040) and anti-rabbit IgG (G21234) were purchased from Invitrogen and diluted at a 1:3000 ratio. Protein bands were detected using SuperSignal^TM^ West Pico PLUS Chemiluminescent Substrate (34580, Thermo Scientific) and FUSION SOLO (Vilber Lourmat, France), and the intensity of the protein bands was quantified using ImageJ 1.54k software.

### 3.13. Production of Reconstructed Skin Tissues

In Korea, some companies are involved in the production of high-quality reconstructed skin tissues and analyses using these tissues, with the objective of making advanced models accessible to the wider scientific community. COSEED BIOPHARM Co., LTD Jeju Branch handles high-quality reconstructed skin tissues composed of normal human melanocytes (NHMs), normal human keratinocytes (NHKs), and fibroblasts. Reconstructed skin tissues were produced by COSEED BIOPHARM Co., LTD Jeju branch (Cheongju, Korea), according to the method described by Ko et al. [61]. Dermal equivalents were obtained after contraction at 37 °C during 5 d of incubation with a mixture containing bovine type I collagen and fibroblasts. NHKs and NHMs were co-seeded at concentrations of 2 × 10^5^ and 2 × 10^4^ cells/well, respectively, on the top of the shrunken dermal equivalent. The culture was then immersed in a co-culture growth medium (CGM) comprising 80% keratinocyte growth medium containing 1.5 mM calcium and 20% melanocyte growth medium for three days to facilitate monolayer formation (immersion phase). In the post-immersion phase, cultures were raised to an air–liquid interface and maintained for at least 12 d for stratification and differentiation of the keratinocytes in CGM supplemented with 50 mg/mL L-ascorbic acid and 10 ng/mL epidermal growth factor.

### 3.14. Hematoxylin and Eosin Staining for Reconstructed Skin Tissues

H&E staining was performed following the modified method described by Ko et al. [61]. Tissues were treated with test samples (500 μg/mL) at 20 μL/1 time for 2 d and incubated for 5 d. Tissues were fixed in 10% neutral formaldehyde for 24 h and transferred to an Optimal Cutting Temperature compound to prepare frozen blocks. The tissues were sectioned at 12 μm for tissue slides. Tissue slides were stained with hematoxylin solution for 2 min, washed, and subsequently stained with Eosin Y solution for 3 min. Tissue slides were dehydrated with alcohol, cleared with xylene, and mounted with Canada balsam. Tissue morphology was examined, and positively stained areas were quantified using ImageJ software.

### 3.15. Immunofluorescence Staining for Reconstructed Skin Tissues

Frozen block slides of reconstructed skin tissues treated with test samples were prepared as described in Section 3.14. Tissue slides were covered with an anti-goat serum-blocking buffer and subsequently incubated overnight with primary antibodies (Filaggrin (1:100, Santacruz) and AQP3 (1:200 Santacruz)) at 4 °C. After washing the primary antibodies with DPBS, tissues were incubated with secondary antibodies (goat anti-mouse IgG Alexa Fluor 488 (1:500) and goat anti-rabbit IgG Alexa Fluor 555 (1:500); Abcam, Cambridge, UK) at 25 °C for 1 h. Tissue slides were washed with DPBS and mounted with a mounting medium containing DAPI. Protein expression in tissues was examined, and positively stained areas were quantified using ImageJ software.

### 3.16. Peptide Profiling

The sample was desalted using a Sep-Pak C18 cartridge. The molecular mass of the peptides was determined using a UHPLC Ultimate 3000 (Thermo Fisher Scientific) and a Q-TOF mass spectrometer (TripleTOF 5600+; AB Sciex, Toronto, ON, Canada). Peptide profiling data analysis was performed using protein pilot v5.0 software (AB Sciex). An ACQUITY UPLC BEH C18 column (130 Å, 1.7 µm, 2.1 × 50 mm, Waters Corporation, Milford, MA, USA) was used at a column temperature of 50 °C and a flow rate of 0.3 mL/min. The mobile phases consisted of distilled water with 0.1% formic acid (A) and acetonitrile with 0.1% formic acid (B): 0 min, 1% B; 0–5 min, 1% B; 5–95 min, 50% B; 95–100 min, 100% B; 100–105 min, 100% B; 105–106 min, 1% B; 106–120 min, 1% B.

### 3.17. Statistical Analyses

All data were generated in triplicate and are presented as means ± standard deviations. A Kruskal-Wallis test was conducted to compare the data, followed by Tukey’s post hoc test using GraphPad Prism 10 software (GraphPad Software, San Diego, CA, USA). Statistical significance was defined as *p* < 0.05.

## 4. Conclusions

Convergence science is evolving at the intersection of several fields in human health. Cosmeceuticals, representative convergence science, have emerged as a substantial area of interest in skin health. Advances in oceanographic science and technology have revealed numerous natural marine-derived product candidates for use as cosmeceuticals. Additionally, sophisticated technologies are being developed to accurately and rapidly assess the potential of these natural products. Furthermore, ongoing efforts are being made to develop environmentally sustainable industries, including the utilization of by-products. This study aimed to explore the potential cosmeceutical use of *H. discus*, a marine mollusk, especially its visceral parts as marine by-products. We confirmed the moisturizing effects of the hydrolysate fraction of the visceral tissue of *H. discus* in HDF and HaCaT keratinocytes by regulating collagen synthesis and HA production. Finally, the moisturizing effects were further validated in reconstructed human skin tissue, an advanced in vitro model, which showed increased levels of AQP3 and filaggrin. In conclusion, these results indicated that the hydrolysate fraction of *H. discus* has potential moisturizing properties and can be used as a cosmeceutical.

## Figures and Tables

**Table 1:** Extraction yield and the proximate composition of four hydrolysates from *H. discus* viscera.

Hydrolysates	Yield (%)	Proximate Composition (%)		
		Protein	Polysaccharide	Total Polyphenol
Alcalase	88.01 ± 0.53 ^a^	38.77 ± 0.45 ^b^	6.69 ± 0.37 ^bc^	2.40 ± 0.01 ^a^
Bromelain	74.21 ± 2.53 ^bc^	37.70 ± 0.26 ^c^	7.99 ± 1.03 ^ab^	2.07 ± 0.11 ^b^
Flavourzyme	70.84 ± 2.11 ^c^	33.20 ± 0.25 ^d^	8.29 ± 0.59 ^a^	2.14 ± 0.06 ^b^
Neutrase	76.21 ± 0.27 ^b^	42.42 ± 0.16 ^a^	6.15 ± 0.50 ^c^	2.32 ± 0.06 ^a^

Values are expressed as the mean ± SD of triplicate experiments. Different lowercase letters indicate significant differences for each experiment.

**Table 2:** Antioxidant activities of four hydrolysates from *H. discus* viscera.

Hydrolysates	Scavenging Activity (IC_50_, mg/mL)		
	DPPH Radical	Hydrogen Peroxide	ABTS Radical
Alcalase	0.81 ± 0.03 ^a^	0.36 ± 0.07 ^b^	0.31 ± 0.00 ^b^
Bromelain	1.11 ± 0.16 ^a^	0.49 ± 0.06 ^ab^	0.34 ± 0.00 ^a^
Flavourzyme	0.88 ± 0.07 ^a^	0.51 ± 0.02 ^a^	0.33 ± 0.00 ^a^
Neutrase	1.19 ± 0.46 ^a^	0.45 ± 0.04 ^ab^	0.31 ± 0.01 ^b^

Values are expressed as the mean ± SD of triplicate experiments. Different lowercase letters indicate significant differences for each experiment.

**Table 3:** Primer information.

Gene	Sequence	Primer
COL1A1	5’-AGCCCTGGTGAAAATGGAGC-3’	Sense
	5’- TCATTTCCACGAGCACCAGC-3’	Antisense
COL1A2	5’-GGCCCTCAAGGTTTCCAAGG-3’	Sense
	5’-CACCCTGTGGTCCAACAACTC-3’	Antisense
COL3A1	5’-TTGAAGGAGGATGTTCCCATCT-3’	Sense
	5’-ACAGACACATATTTGGCATGGTT-3’	Antisense
HAS1	5’-CCACCCAGTACAGCGTCAAC-3’	Sense
	5’-CATGGTGCTTCTGTCGCTCT-3’	Antisense
HAS2	5’-GTCGAGTTTACTTCCCGCCA-3’	Sense
	5’-ATCACACCACCCAGGAGGAT-3’	Antisense
HAS3	5’-GATTTCCTTCCTGAGCAGCG-3’	Sense
	5’-TGTTGCGGTACATGCCCAAG-3’	Antisense
AQP3	5’-TGCAATCTGGCACTTCGC-3’	Sense
	5’-GCCAGCACACACACGATAA-3’	Antisense
Filaggrin	5’-GGCTAAGTGAAAGACTTGAAGAGA-3’	Sense
	5’-AATAGACTATCAGTGGTGTCATAGG-3’	Antisense
β-actin	5’-CACTGTGCCCATCTACG-3’	Sense
	5’-CTTAATGTCACGCACGATTTC-3’	Antisense

## Data Availability

The original contributions presented in the study are included in the article; further inquiries can be directed to the corresponding author.
